# Impact of Native Form Oat β-Glucan on the Physical and Starch Digestive Properties of Whole Oat Bread

**DOI:** 10.3390/foods11172622

**Published:** 2022-08-29

**Authors:** Han Hu, Huihui Lin, Lei Xiao, Minqi Guo, Xi Yan, Xueqian Su, Lianliang Liu, Shangyuan Sang

**Affiliations:** 1 Department of Food Science and Engineering, Ningbo University, Ningbo 315832, China; 2 Department of Food Science and Technology, Virginia Polytechnic Institute and State University, Blacksburg, VA 24061, USA

**Keywords:** low glycemic index, β-glucan, dough properties, bread quality, starch digestion

## Abstract

To investigate the effect of oat bran on bread quality and the mechanism of reducing the glycemic index (GI) of bread, wheat bran (10%, w/w, flour basis), oat bran (10%), and β-glucan (0.858%) were individually added to determine the expansion of dough, the specific volume, texture, color, GI, starch digestion characteristics, and α-amylase inhibition rate of bread. The results showed that the incorporation of wheat bran and oat bran both reduced the final expanded volume of the dough, decreased the specific volume of the bread, and increased the bread hardness and crumb redness and greenness values as compared to the control wheat group. The above physical properties of bran-containing bread obviously deteriorated while the bread with β-glucan did not change significantly (*p* < 0.05). The GI in vitro of bread was in the following order: control (94.40) > wheat bran (69.24) > β-glucan (65.76) > oat bran (64.93). Correspondingly, the oat bran group had the highest content of slowly digestible starch (SDS), the β-glucan group had the highest content of resistant starch (RS), and the control group had the highest content of rapidly digestible starch (RDS). For the wheat bran, oat bran, and β-glucan group, their inhibition rates of α-amylase were 9.25%, 28.93%, and 23.7%, respectively. The β-glucan reduced the bread GI and α-amylase activity by intertwining with starch to form a more stable gel network structure, which reduced the contact area between amylase and starch. Therefore, β-glucan in oat bran might be a key component for reducing the GI of whole oat bread.

## 1. Introduction

Diabetes is a metabolic disorder of carbohydrates, proteins, and fats caused by insufficient insulin secretion or insensitivity in the body [1]. According to the latest data released by the International Diabetes Federation, about 537 million adults worldwide were living with diabetes in 2021 and this number is expected to increase continuously in the next 10–25 years [2]. In addition, China has the largest number of diabetics in the world and the incidence rate is still on the rise, becoming a significant public health problem affecting people’s physical and mental health [2]. Recent studies have found that dietary intervention with a low glycemic index (GI) can significantly reduce the drug dosage of diabetics, which can finally reduce the risk of diabetes as well as related complications. This has been recognized as a low-cost yet effective way to control diabetes [3]. Therefore, the development of low GI food products is of great significance for diabetic patients.

Oat bran is a common by-product of the grinding process of whole oats. It is low in digestible carbohydrates and rich in β-glucans, polyphenols, and flavonoids. It was reported that each gram of β-glucan in oat bran can reduce the area under the glucose curve by 4.35% (r = 0.507, *p* = 0.0008, *n* = 40) and the peak by 6.57% (r = 0.582, *p* < 0.0001) [4,5], probably owing to the high viscosity of oat β-glucan which can form a sticky solution in the intestinal tract, reduce the contact of food and digestive enzymes, and delay gastric emptying and the formation of blood glucose. Zhang et al. [6] pointed out that β-glucan enhanced the viscosity of the digestive tract through its own aggregation and intertwining and overlapping with starch, thus restricting water mobility and reducing the contact between digestive enzymes and starch, glucose, and small intestinal microvilli. This means that the long-term consumption of oat bran can decrease the level of blood glucose and cholesterol, significantly contributing to the prevention of cardiovascular and cerebrovascular diseases [7]. In addition, oat β-glucan can inhibit glucose transport by down-regulating the glucose transporter in small intestinal epithelial cells. Oat bran polyphenols are secondary metabolites of oat, including phenolic acids and flavonoids. It was found that oat bran polyphenols can significantly reduce fasting glucose content and increase superoxide dismutase activity in diabetic mice, suggesting that the mechanism of reducing the blood glucose of oat polyphenols may be related to their antioxidant and scavenging free radical ability [8]. However, it has not yet been confirmed by research which active ingredient plays a more important role in lowering the GI.

Bread is a kind of nutritious, easy-to-digest, and convenient staple food. With the accelerating pace of life, consumers’ demand for baking products is growing nowadays, and at the same time, they are not willing to sacrifice the health-promoting effects of these products. Traditional bread is usually made of ordinary wheat flour and processed with white sugar, which has a high GI and is unsuitable for diabetics. Previous research has focused on adding low-GI ingredients to bread formulas, such as partially replacing high-gluten flour with wheat bran. However, this will lead to a decrease in the physical quality of the bread. It was reported that β-glucan in oat bran was 2.6 times higher than that in wheat bran [9], suggesting the better performance of oat bran regarding the decrease in blood glucose as compared to wheat bran. Furthermore, oat bran products were proved to possess better quality than other grain bran products. Pavan et al. [10] found that among the biscuits with an added 3–7% (*w*/*w*, flour basis) wheat bran or oat bran, the physical characteristics of oat bran biscuits were better than wheat bran biscuits. The results of Yadav et al. [11] showed that oat bran can increase moisture content (*p*  ≤  0.05) and reduce oil absorption during frying, while wheat bran has the opposite effect. These studies suggested that oat bran has greater potential than wheat bran in developing high-quality baked foods with low GI.

In order to verify that oat bran bread has better physical qualities and lower GI than wheat bran bread and explore the main substances in oat bran that play a role in reducing GI, in this study, the effects of oat bran and oat β-glucan on the physical and starch digestion properties of bread were investigated by adding oat bran or oat β-glucan to a bread formula and comparing it with regular bread and bread with added wheat bran. It will provide a theoretical basis for developing oat bran food and enriching varieties of low GI food.

## 2. Materials and Methods

### 2.1. Materials

High-gluten wheat flour containing 14.38% protein, 72.19% starch, 0.45% ash, and 12.83% moisture was supplied by the Henan Wudeli Flour Group Corp. (Zhoukou, China). wheat bran with an average particle size of 425 μm (*Triticum aestivum* L.) containing 13.64% moisture, 15.83% protein, and 0.53% ash was obtained from Henan Jinyuan Grain and Oil Co., Ltd. (Zhengzhou, China). Oat bran with an average particle size of 425 μm (*Avena sativa* L.) containing 8.64% moisture, 20.80% protein, 8.58% β-glucan [12], and 2.25% ash was purchased from Zhangjiakou Jianjun oat Food Co., Ltd. (Zhangjiakou, China). Instant dry yeast (*Saccharomyces cerevisiae*) was obtained from Angel yeast Co., Ltd. (Yichang, China). β-Glucan with an average molecular weight of 5300 Da was purchased from Ebara Biotechnology Co., Ltd. (Guangzhou, China). All the other reagents used in this study were of analytical grade and obtained from Sinopharm (China National Medicines Corporation Ltd., Shanghai, China).

### 2.2. Determination of Dough Properties

#### 2.2.1. Dough Production

The dough production was conducted according to the method reported by Sang et al., with slight modifications [13]. The water absorption of the dough and the mixing time of the dough should be measured by farinograph and mixograph, but due to the poor limited experimental conditions in our lab, it could only be estimated according to the report of Sang et al. and the experience and quality of the final bread. According to Table 1, the ingredients of each group were put in a dough mixer and kneaded at a low speed (the first gear of the dough mixer) for 4 min, then at a high speed (the second gear of the dough mixer) for 15 min, until the surface of the dough was smooth and could be pulled into a gluten film by hand.

#### 2.2.2. Extension Test of the Dough

The extension test of the doughs was conducted according to the method reported by Sang et al. with slight modifications [13]. The doughs prepared for the extension test were yeast-free strips with a length of 65 mm and a cross-section of 4 × 4 mm. They were coated with a small amount of oil before measurement. The dough extension curves were measured by TA.XT Plus Texture Analyzer (Stable Micro System Co., Ltd., Godalming, Surrey, UK) equipped with Kieffer dough and gluten extensibility rig. Specifically, dough strips were stretched from 10 mm to 60 mm at a rate of 3.30 mm/s under the tension mode. The distance (mm) and force (mN) were used as the x-axis and y-axis, respectively. The force and the distance corresponding to the vertex of the curve were defined as the resistance (mN) and extension (mm), respectively.

#### 2.2.3. Expansion Volume of the Dough 

The method described by Sang et al. [13] was used to measure the expansion volume of the dough. To be specific, 50 g of dough was put into the bottom of a 250 mL sterilized measuring cylinder and fermented at 37 °C and 80% relative humidity for 100 min. The dough volume gradually expanded during the fermenting process, and the difference between the final and initial dough volume was defined as its expansion volume. 

### 2.3. Determination of Bread Quality

#### 2.3.1. Bread Making

The bread-making process was slightly modified from the method described by Sang et al. [13]. The kneaded dough was divided into 5 parts (150 g each). After shaping, the doughs were put in a toast box (151 × 67 × 67 mm) and fermented at 37 °C and 80% relative humidity for 90 min. After baking for 15 min in an oven at 215 °C (top temperature) and 190 °C (bottom temperature), the bread was immediately taken out and cooled at room temperature for 2 h. Its quality parameters were determined within 12 h. 

#### 2.3.2. Specific Volume of the Bread

The method reported by Ozmutlu et al. was applied to measure the specific volume of the bread with slight modifications [14]. The rapeseed replacement method was used to measure the bread volume. Fill a 1000 mL beaker with rapeseed, scrape it flat with a ruler, and pour it into a measuring cylinder to measure the volume. The cooled bread sample was weighed and put into the above-mentioned 1000 mL beaker, followed by the addition of rapeseed until the bread sample was covered and the beaker was filled. Scraping it flat with a ruler, the volume of remaining rapeseed was the bread volume. The specific volume of bread was the ratio of the volume to the mass of the bread after cooling.

#### 2.3.3. Texture Analysis of the Bread 

The texture analysis was conducted according to the method reported by Ozmutlu et al. with slight modifications [14]. The cooled bread was cut into even slices with 24-mm thickness, and the textures of intermediate slices were analyzed by TA.XT Plus Texture Analyzer with a P/25 probe. The trigger force was 5 g. The pre-test, in-test, and post-test speeds were set at 3 mm/s, 1 mm/s, and 5 mm/s, respectively. The sample was compressed to 40% of its original height. The two compression intervals were 5 s, and the probe trigger force was 5 g. The samples were measured in 5 replicates, and the mean and standard deviation were calculated.

#### 2.3.4. Moisture Content of the Bread

The method reported by Nilufer et al. was used, with slight modifications [15]. The moisture content of the bread was determined by the direct drying method. An amount of 2~10 g (accurate to 0.0001 g) of crushed bread samples were weighed in the weighing bottle. The samples were placed in the oven with the temperature pre-adjusted to 101~105 °C, and the cap of the weighing bottle was tilted against the bottle edge and dried for 2~4 h. Then, the weighing bottle was taken out with the lid and put into the dryer to cool for about 0.5 h. After the temperature drops to room temperature, the weighing bottle was weighed, then put into the oven again for drying for about 1 h, taken out, and put into a dryer to cool for 0.5 h before weighing. The operation was repeated until the weight was constant (the weight difference between the two weights should not exceed 2 mg).

#### 2.3.5. Color Analysis of the Bread

The color analysis was measured according to the method reported by Nilufer et al. with slight modifications [15]. The L*, a*, and b* values of the bread crust and core were determined by the Hunter Lab colorimetric system (Model NR110, Shenzhen 3nh Technology Co., Ltd., Shenzhen, China). The L* value represents the lightness of the bread sample. The larger L* value indicates higher brightness. The a* value represents the green–red degree of the bread sample, with −a* representing green and +a* representing red. The b* value represents the blue–yellow degree of the bread sample, with −b* representing blue and +b* representing yellow. The colorimeter was preheated for 30 min before use. After whiteboard calibration, the lens was aimed at the top surface and bread core of the bread slice, and the test button was pressed to read the value. The measurement was repeated 5 times.

### 2.4. Evaluation of Low GI efficacy

#### 2.4.1. Estimated Glycemic Index In Vitro 

The method reported by Yousif et al. [16] was slightly modified for the determination of the estimated glycemic index in vitro. Crumbled bread core (0.5 g) was accurately weighed in a 50 mL conical flask with a lid, and 1 mL porcine pancreatic α-amylase solution (50 U /mL, 0.2 mol/L pH = 7.0 phosphoric acid buffer) was added for a reaction for 15 s to simulate oral digestion. A total of 5 mL pepsin (4 mg/mL, 0.02 mol/L HCl) was then added and stirred in a 37 °C water bath at 130 r/min for 30 min to simulate gastric digestion. NaOH solution (5 mL, 0.02 mol/L) was used to neutralize the acid, then phosphoric acid buffer (25 mL, 0.2 mol/L pH = 6.0) and mixed enzyme solution (5 mL, porcine pancreatic α-amylase 2 mg/mL, glucosidase 28 U/mL, 0.2 mol/L pH = 6.0 phosphoric acid buffer) were added to simulate intestinal digestion. At 0, 20, 90, 120, 150, and 180 min, a small amount of digestive solution (0.5 mL) was taken from digesta and placed in 5 mL centrifuge tubes, respectively. After adding 2 mL of anhydrous ethanol solution to each centrifuge tube, the mixture was centrifuged at 1753× *g* for 10 min. The glucose content of the supernatant was determined by a glucose kit The hydrolysis rate (HR) of the sample was calculated as the ratio of the glucose content calculated above to the total starch content in the original sample and multiplied by a coefficient of 0.9. The hydrolysis rate curve can be obtained by plotting HR against hydrolysis time. The area under the hydrolysis rate curve (AUC) was calculated by Origin software, and the hydrolysis index (HI, %) of the sample was calculated according to Formula (1).

(1) $$\mathrm{HI}=\frac{{\mathrm{AUC}}_{1}}{{\mathrm{AUC}}_{0}}\times 100\%$$

where AUC_1_ and AUC_0_ were the areas under the hydrolysis rate curve of starch in the sample and the standard white bread, respectively.

Finally, the estimated glycemic index (eGI) was obtained according to Formula (2).

(2) eGI=0.862HI+8.1981

#### 2.4.2. In Vitro Starch Digestion Characteristics

The method reported by Yousif et al. [16] was slightly modified for the determination of starch digestion characteristics in vitro. At 0, 20, 120, and 180 min, a small amount of digestive solution (0.5 mL) was taken from digesta and placed in 5 mL centrifuge tubes, respectively. After adding 2 mL of anhydrous ethanol solution to each centrifuge tube, the mixture was centrifuged at 1753× *g* for 10 min. The glucose content of the supernatant was determined by a glucose kit. The contents of rapidly digestible starch (RDS), slowly digestible starch (SDS), and resistant starch (RS) were calculated as follows:

(3) $$R_{\mathrm{RDS}}/\%=\frac{m_{20}-m_{0}}{m_{180}}\times 100\;$$

(4) $$R_{\mathrm{SDS}}/\%=\frac{m_{120}-m_{20}}{m_{180}}\times 100$$

(5) $$R_{\mathrm{RS}}/\%=1-R_{\mathrm{RDS}}-R_{\mathrm{SDS}}$$

where *m*_0_ was the mass (mg) of free glucose in the sample before enzymatic hydrolysis; *m*_20_, *m*_120_, and *m*_180_ were the mass (mg) of glucose generated after enzymatic hydrolysis for 20 min, 120 min, and 180 min, respectively.

#### 2.4.3. α-Amylase Inhibition Rate

The method reported by Lu et al. [17] was used. Fifty grams of bread samples in each group were added to 400 mL of NaCl solution (0.25 mol/L). The mixture was stirred at room temperature for 2 h and then filtered. The collected filtrate was heated in a 70 °C water bath for 30 min and centrifuged at 1753× *g* for 15 min to collect the supernatant. α-amylase solution (1 mg/mL) was added to the supernatant at a ratio of 1:1 (*v*/*v*) and reacted in a 37 °C water bath for 10 min, followed by the addition of 2 mL starch solution further incubation at the same temperature for 5 min. The mixture was subjected to the boiling water bath for 5 min to deactivate the α-amylase activity, after which 1 mL of iodine potassium iodide solution was added. After cooling, the obtained solution was diluted, and the absorbance value was measured at 535 nm (UV-1900 type UV spectrophotometer, Shimadzu Company, Kyoto, Japan), which was recorded as *A*_1_. In the control group, 0.5 mL distilled water was used to replace bread water extract, and the absorbance values were recorded as *A*_2._ In the blank group, 1 mL distilled water was used to replace α-amylase solution and bread water extract; the absorbance values were recorded as *A*_0_.

(6) $$Inhibition\;rate\;of\;\alpha -amylase=\frac{\left(A_{2}-A_{0}\right)-\left(A_{1}-A_{0}\right)}{A_{2}-A_{0}}\times 100\%$$

where *A*_0_ was the absorbance value of the mixture after diluting, *A*_1_ was the absorbance value of the control group in which 0.5 mL distilled water was used to replace bread water extract, and *A*_2_ was the absorbance value of the blank group in which 1 mL distilled water was used to replace α-amylase solution and bread water extract.

### 2.5. Statistical Analysis

The experiment was carried out 3–6 times. The data are reported as the mean  ±  SD. The statistical analysis was performed by one-way analysis of variance, employing SPSS, version 18.5 (IBM Corp., Armonk, NY, USA).

## 3. Results and Discussion

### 3.1. Effects of Different Additives on the Extensibility and Expansion Volume of Dough

**Figure 1:**
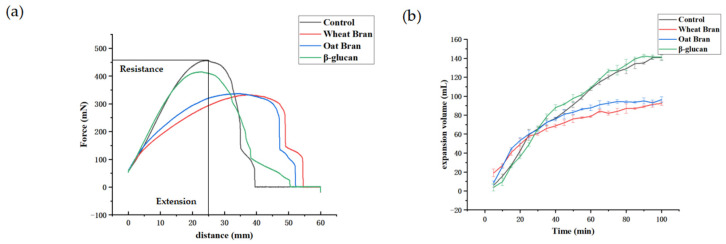
Effects of different additives on the tensile properties (**a**) and expansion curves (**b**) of the dough.

The resistance is a sign of the longitudinal elasticity of the dough, which indicates the toughness of the dough [18]. As seen in Figure 1a, the wheat and oat bran reduced the maximum resistance of the dough compared to the control group. The reason might be that the bran loosens the gluten structure of the mixed dough, which reduces the resistance [19,20,21]. The more significant reduction in the resistance of the dough made by wheat bran may be ascribed to the higher fiber content in the wheat bran, resulting in a looser gluten structure. Compared with the control group, β-glucan significantly decreased the resistance of the dough. Therefore, replacing wheat bran with oat bran or β-glucan is a promising approach to balance the nutritional value of low GI bread with the resistance index of the dough.

The expansion volume of dough is an important parameter to characterize a series of physical and chemical changes during the fermentation process and the quality of flour baking. As shown in Figure 1b, the dough volume of the control group and the β-glucan group continued to increase as time progressed. The dough volume of the wheat bran group and the oat bran group showed an initial increase and then leveled off, indicating the reduced air holding capacity caused by the bran addition, and β-glucan has little effect on the expansion volume of the dough compared to the control group. The continuous gluten network is a necessity for the formation and stability of the air cells in this matrix. The crude fiber in the wheat and the oat bran can disrupt the continuity of the gluten network, leading to the rupture of air cells and the reduction in the expansion volume of the dough [22,23]. Another possible reason is that the bran tends to absorb more water from the dough system, resulting in insufficient hydration and the weakening of the gluten film as well as the merging of bubbles within the dough. This can, finally, cause the decreased gas holding capacity of the dough and the reduced volume of the bran bread.

### 3.2. Effects of Different Additives on Bread Quality

#### 3.2.1. Specific Volume and Texture Properties of Bread

The specific volume and texture properties of the bread are shown in Table 2. The addition of wheat and oat bran reduced the specific volume of the bread. It may be that bran contains more insoluble dietary fiber particles, disrupting the structure of the gluten protein [24], which hinders the formation of the gluten network in the dough and reduces the expansion volume of the dough and the volume of bread. As a hydrophilic colloid, β-glucan has a reinforcing effect on gluten (Figure 1a), increasing the expansion volume of the dough, thereby increasing the specific volume of the bread [25]. 

According to Table 2, adding wheat bran, oat bran, and β-glucan significantly affected the textural properties of the bread. Compared with the control group, wheat and oat bran significantly increased bread hardness and chewiness and decreased resilience [26]. The crude fiber content of wheat bran is higher than that of oat bran [10], which might be a reason for the harder and less elastic texture of the bread made by the wheat bran. As a soluble dietary fiber, β-glucan has a high water-binding capability, contributing to better hydration and the full formation of the gluten network in the dough matrix. This helped to improve the resilience and recovery while reducing the hardness of bread [27].

#### 3.2.2. The Moisture Content and Color of Bread Crust and Crumb

As can be seen from Table 3, wheat bran, oat bran, and β-glucan all considerably increased the moisture content of bread. This may be attributed to the high water-binding capability of some dietary fibers in bran such as β-glucan, which helped to retard the water diffusion and increase the water retention in the internal microstructure of bread [28,29].

Overall, compared with the control group, the incorporation of wheat bran and oat bran changed the color of bread to different degrees. Adding wheat bran and oat bran increased the a* and b* values of the crumb (*p* < 0.05). It is possible that various pigments in the oat and wheat bran such as chlorophyll, carotene, and lutein had the possibility to darken the crumb color, leading to the increase in a* or b* in the crumb of the wheat and oat bran groups as compared to that in the β-glucan group [30]. The other reason was that the wheat or oat bran was richer in amino acids than the β-glucan, which enhanced the Maillard reaction and led to a darker crumb color. Compared with the change in the color of the crumb, there was no significant change in the crust. This was because, in addition to the Maillard reaction, the caramelization reaction that took place during baking was also the main cause of the brown crust. The glycosidic bonds in the fiber from the wheat, oat bran, and oat β-glucan were broken into oligosaccharides and monosaccharides and caramelized under high temperature [31]. 

### 3.3. Effects of Different Additives on Bread GI

#### 3.3.1. eGI In Vitro

**Figure 2:**
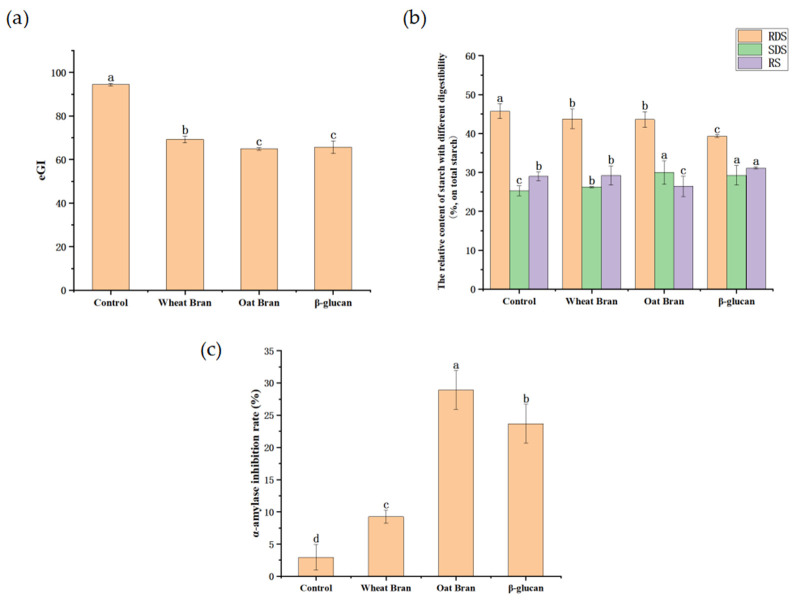
(**a**) Effects of different additives on the estimated glycemic index (eGI) of bread; (**b**) effects of different additives on the relative content of starch with different digestibility in the breads; (**c**) effects of different bread water extracts on the inhibition rate of α-amylase. Note: Different lowercase letters among the data in the same index in each subfigure indicate significant differences (*p* < 0.05).

The eGI is shown in Figure 2a. Adding wheat bran, oat bran, and β-glucan all significantly reduced the bread GI. The eGI of the β-glucan (65.76) bread was slightly higher than that of the oat bran (64.93), although they had similar eGI. The results were generally consistent with the findings of Kim et al. The eGI is inversely correlated with β-glucan concentration in heated oat flour slurry [32]. The possible reason is that the β-glucan in oat bran reduced the content of RDS in bread by inhibiting the activity of pancreatic α-amylase, increasing the content of SDS and reducing the fluctuation of postprandial blood glucose [33,34,35,36]. This hypothesis was also verified in the following experiments.

#### 3.3.2. In Vitro Digestion Properties of Starch

RDS is rapidly digested and absorbed by the human body, which can cause a rapid rise in blood glucose and is not beneficial for health. SDS, on the contrary, can be digested and absorbed by the body at a significantly slower rate. It can continuously release energy to the human body and thus prolong satiety. RS cannot be digested and utilized by the small intestine and has no significant impact on postprandial blood glucose [37]. As seen in Figure 2b, the addition of wheat bran, oat bran, and β-glucan considerably reduced the RDS content and increased the SDS content in bread. Compared with the control group, β-glucan significantly increased RS content, while oat bran decreased RS content, but the wheat bran group had no significant effect on RS content. Compared with the oat bran group, β-glucan bread contained higher RS content and similar SDS content. β-glucan is a cell wall component of raw plant materials that is viscous and difficult to be digested and absorbed [38]. It may have increased the viscosity of the entire system, which slowed down the digestion process, prolonged gastric emptying, and prevented food from reaching the small intestine for a short time. The high viscosity of β-glucan could also promote its binding with other bread fractions and reduce the interaction between amylase and starch, which retarded starch degradation [24,39,40].

#### 3.3.3. α-Amylase Inhibition Rate

α-amylase is a critical enzyme that affects the digestion and absorption of starch in food. Therefore, this experiment evaluated the low GI efficacy of bread by measuring the inhibition rate of α-amylase by extracts from the different breads. As shown in Figure 2c, wheat bran, oat bran, and β-glucan had specific inhibitory effects on the α-amylase inhibition rate, among which oat bran had the most potent ability to inhibit α-amylase activity. The inhibition rate of α-amylase in the β-glucan group (23.7%) was larger than half (14.46%) of that of the oat bran group, indicating that β-glucan in oat bran played a significant role in inhibiting the activity of α-amylase. Previous studies have shown that β-glucan can intertwine with starch to form a more stable gel network structure, which reduces the contact area between amylase and starch, thus reducing the action of α-amylase [41]. Other active substances in oat bran also play a part in the inhibition of α-amylase [6]. For example, phenolic acids contain phenolic hydroxyl and carboxyl groups, which can interact with proteins to affect the activity of enzymes.

In this study, the contents of RDS, SDS, and RS in bread were determined by simulating in vitro digestion. The results showed that β-glucan bread contained higher RS content and similar SDS content than oat bran bread. This was mainly because β-glucan increased the viscosity of the digestive system, thus slowing down the digestion speed. However, in addition to β-glucan, other substances may also have some influence on the digestion characteristics of starch in oat bran. Finally, the SDS content of oat bran was similar to that of the β-glucan bread, while the RS content of oat bran was lower than that of the β-glucan bread. It had been reported that in addition to the inhibitory effect of β-glucan on α-amylase in oat bran [6], the phenolic acids in oat bran also have a certain inhibitory effect on α-amylase, so the inhibitory rate of oat bran bread was higher than that of oat β-glucan bread. After combining the above two conditions, the eGI of β-glucan bread (65.76) was slightly (*p* > 0.05) higher than that of oat bran (64.93) although they had similar eGI.

## 4. Conclusions

Compared with the control group, adding 10% wheat bran or 10% oat bran both reduced the extension resistance of the dough as well as the expansion ability, specific volume, and softness of the bread. The final expanded volume of the dough decreased by 31.67% and 34.36%, the specific volume decreased by 16.57% and 10.77%, the hardness increased by 38.13% and 40.89%, the crumb’s a* value increased by 80.92% and 87.31%, and the b* value increased by 21.53% and 31.69%. However, they both reduced the RDS content and increased the SDS content and the inhibition rate of α-amylase, thereby reducing the GI of bread. Compared with the oat bran group, adding oat β-glucan reduced the GI while maintaining the excellent sensory properties of the bread. Therefore, β-glucan is the component in the oat bran that significantly contributes to the low GI values in the whole oat bread.

## Figures and Tables

**Table 1:** Ingredients in the test doughs.

Ingredients	Control (g)	Wheat Bran (g)	Oat Bran (g)	β-Glucan (g)
Wheat flour	500 (100%)	500 (100%)	500 (100%)	500 (100%)
Water	300 (60%)	330 (66%)	330 (66%)	300 (60%)
Sugar	40	40	40	40
Dry yeast	9	9	9	9
NaCl	4	4	4	4
Wheat bran	-	50 (10%)	-	-
Oat bran	-	-	50 (10%)	-
β-Glucan	-	-	-	4.29 (0.858%)

Note: The recipe used by the control bread was the basic formula.

**Table 2:** Effects of different additives on the specific volume and texture properties of bread.

Group	Specific Volume (cm^3^/g)	Hardness (N)	Resilience	Chewiness	Recovery
Control	3.62 ± 0.01 ^b^	3.05 ± 0.03 ^b^	0.69 ± 0.01 ^b^	1.37 ± 0.06 ^b^	0.29 ± 0.00 ^b^
Wheat bran	3.02 ± 0.08 ^d^	4.93 ± 0.35 ^a^	0.67 ± 0.01 ^c^	1.83 ± 0.24 ^a^	0.27 ± 0.00 ^c^
Oat bran	3.23 ± 0.02 ^c^	5.16 ± 0.37 ^a^	0.66 ± 0.01 ^c^	1.98 ± 0.12 ^a^	0.27 ± 0.01 ^c^
β-Glucan	3.82 ± 0.04 ^a^	2.85 ± 0.25 ^b^	0.71 ± 0.01 ^a^	1.12 ± 0.09 ^c^	0.32 ± 0.00 ^a^

Note: Different lowercase letters among the data in the same column indicate significant differences (*p* < 0.05).

**Table 3:** Effects of different additives on the moisture content and color of bread crust and crumb.

Group	Crust				Crumb			
	Moisture Content (%)	L*	a*	b*	Moisture Content (%)	L*	a*	b*
Control	23.45 ± 0.32 ^c^	45.16 ± 2.04 ^a^	18.80 ± 0.84 ^a^	33.31 ± 1.54 ^a^	42.57 ± 0.18 ^b^	65.11 ± 0.22 ^a^	0.33 ± 0.08 ^c^	10.60 ± 0.48 ^c^
Wheat bran	28.03 ± 1.00 ^a^	44.36 ± 0.96 ^a^	19.42 ± 0.59 ^a^	33.34 ± 0.71 ^a^	45.90 ± 0.54 ^a^	63.10 ± 0.72 ^ab^	1.73 ± 0.16 ^b^	13.51 ± 0.79 ^b^
Oat bran	26.14 ± 0.38 ^b^	40.72 ± 0.24 ^b^	19.18 ± 0.17 ^a^	29.30 ± 1.39 ^b^	45.65 ± 0.26 ^a^	62.93 ± 1.65 ^ab^	2.60 ± 0.16 ^a^	15.40 ± 0.65 ^a^
β-Glucan	27.48 ± 0.89 ^ab^	45.07 ± 1.07 ^a^	19.00 ± 0.68 ^a^	33.99 ± 0.59 ^a^	45.62 ± 0.50 ^a^	61.56 ± 1.47 ^b^	0.43 ± 0.24 ^c^	10.95 ± 0.69 ^c^

Note: Different lowercase letters among the data in the same column indicate significant differences (*p* < 0.05). The L* value represents the lightness of the bread sample. The larger the L* value, the whiter the sample. The a* value represents the green–red degree of the bread sample, with −a* representing green and +a* representing red. The b* value represents the blue–yellow degree of the bread sample, with b* representing blue and +b* representing yellow.

## Data Availability

Data is contained within the article.

## Glossary

eGI	estimated glycemic index
GI	glycemic index
RDS	rapidly digestible starch
RS	resistant starch
SDS	slowly digestible starch
